# Novel Interactome of *Saccharomyces cerevisiae* Myosin Type II Identified by a Modified Integrated Membrane Yeast Two-Hybrid (iMYTH) Screen

**DOI:** 10.1534/g3.115.026609

**Published:** 2016-02-25

**Authors:** Ednalise Santiago, Pearl Akamine, Jamie Snider, Victoria Wong, Matthew Jessulat, Viktor Deineko, Alla Gagarinova, Hiroyuki Aoki, Zoran Minic, Sadhna Phanse, Andrea San Antonio, Luis A. Cubano, Brian C. Rymond, Mohan Babu, Igor Stagljar, Jose R. Rodriguez-Medina

**Affiliations:** * Department of Biochemistry,University of Puerto Rico, San Juan, Puerto Rico 00936-5067; † Center for Molecular Sciences and Research, University of Puerto Rico, San Juan, Puerto Rico 00926; ‡ Department of Biochemistry, Department of Molecular Genetics, Donnelly Centre, University of Toronto, Ontario M5S 3E1, Canada; § Department of Biochemistry, University of Regina, Saskatchewan, Canada; ** Department of Biochemistry, College of Medicine, University of Saskatchewan, Canada; †† School of Medicine, Universidad Central del Caribe, Bayamon, Puerto Rico 00960-6032; ‡‡ Department of Biology, University of Kentucky, Lexington, Kentucky 40506

**Keywords:** Myo1p, proteomics, interactome, cytokinesis, yeast

## Abstract

Nonmuscle myosin type II (Myo1p) is required for cytokinesis in the budding yeast *Saccharomyces cerevisiae*. Loss of Myo1p activity has been associated with growth abnormalities and enhanced sensitivity to osmotic stress, making it an appealing antifungal therapeutic target. The Myo1p tail-only domain was previously reported to have functional activity equivalent to the full-length Myo1p whereas the head-only domain did not. Since Myo1p tail-only constructs are biologically active, the tail domain must have additional functions beyond its previously described role in myosin dimerization or trimerization. The identification of new Myo1p-interacting proteins may shed light on the other functions of the Myo1p tail domain. To identify novel Myo1p-interacting proteins, and determine if Myo1p can serve as a scaffold to recruit proteins to the bud neck during cytokinesis, we used the integrated split-ubiquitin membrane yeast two-hybrid (iMYTH) system. Myo1p was iMYTH-tagged at its C-terminus, and screened against both cDNA and genomic prey libraries to identify interacting proteins. Control experiments showed that the Myo1p-bait construct was appropriately expressed, and that the protein colocalized to the yeast bud neck. Thirty novel Myo1p-interacting proteins were identified by iMYTH. Eight proteins were confirmed by coprecipitation (Ape2, Bzz1, Fba1, Pdi1, Rpl5, Tah11, and Trx2) or mass spectrometry (AP-MS) (Abp1). The novel Myo1p-interacting proteins identified come from a range of different processes, including cellular organization and protein synthesis. Actin assembly/disassembly factors such as the SH3 domain protein Bzz1 and the actin-binding protein Abp1 represent likely Myo1p interactions during cytokinesis.

The *Saccharomyces cerevisiae* myosin type II (Myo1p) is found in the contractile ring that contributes to its function in cell division coupled with chitin synthase 2 driven membrane ingression (Bi *et al.* 1998; VerPlank and Li 2005; Schmidt *et al.* 2002). Myo1p is a large protein (223.6 kDa) that consists of a globular N-terminal head domain [amino acids (aa) 1–800], and a long tail (aa 850–1928). The globular head binds to filamentous actin and ATP. The tail domain has been predicted to adopt a coiled-coil conformation with breaks (May *et al.* 1998). Unexpectedly, Myo1p was found to function in cytokinesis as a tail-only domain (Tolliday *et al.* 2003; Lord *et al.* 2005; Lister *et al.* 2006), suggesting that the N-terminal domain power-stroke function is not an essential feature of Myo1p. Furthermore, a minimum localization domain (MLD) identified in the terminal 1000 aa, which was previously associated with Myo1p oligomerization, appears to provide additional biological activity, and possibly serves as a site for the recruitment of the cytokinesis machinery and/or to signal for cell division (Lister *et al.* 2006). Previous yeast two-hybrid experiments (Drees *et al.* 2001; Wang *et al.* 2012), and TAP-tag protein copurification experiments (Gavin *et al.* 2002) identified multiple putative Myo1p-interacting proteins, but shed little light on the role of the Myo1p tail as a potential platform for the recruitment of proteins that may regulate cytokinesis. Earlier studies relied heavily on the recovery of soluble proteins, which the Myo1p-protein interactions were likely biased against. In this study, we used a modified integrated split-ubiquitin membrane yeast two-hybrid (iMYTH) technique to search for Myo1p interactions with associated proteins (Stagljar *et al.* 1998; Paumi *et al.* 2007; Snider *et al.* 2010, 2013) that may support the function of the C-terminal region of Myo1p at the bud neck during cytokinesis.

## Materials and Methods

### Yeast strains and culture conditions

All yeast strains were grown at 30° in YPD broth while shaking at 225 rpm (Table 1). Artificial bait L40, Myo1 L40 L2, and Myo1 L40 L3 strains were maintained on YPD or YPD +200 μg/ml of G418 with 2% agar medium, and were transferred to synthetic dropout medium without tryptophan to select for retention of prey plasmids during screening. Myo1-GFP strains were maintained in synthetic medium lacking histidine. Strains used in this study are available upon request.

**Table 1:** Strains used in this study

Strain	Genotype	Source
L40	*MATa HIS3 200 trp1-901 leu2-3*, *112 ade2 LYS2*::*(lexAop)_4_-HIS3 URA3*::*(lexAop)_8_-lacZ GAL4*	Stagljar laboratory
Artificial bait in L40 (A0287)	*MATa HIS3 200 trp1-901 leu2-3*, *112 ade2 LYS2*::*(lexAop)_4_-HIS3 URA3*::*(lexAop)_8_-lacZ GAL4 Matα-CD4(TM)*::*(Cub-YFP-lexA-VP16-KanMX)*	Stagljar laboratory
Myo1 L40 L2	*MATa HIS3 200 trp1-901 leu2-3*, *112 ade2 LYS2*::*(lexAop)_4_-HIS3 URA3*::*(lexAop)_8_-lacZ GAL4 MYO1-(Cub-lexA-VP16-KanMX)*	This study
Myo1 L40 L3	*MATa HIS3 200 trp1-901 leu2-3*, *112 ade2 LYS2*::*(lexAop)_4_-HIS3 URA3*::*(lexAop)_8_-lacZ GAL4 MYO1-(Cub-YFP-lexA-VP16-KanMX)*	This study
BY4741	*MATa his3delta1 leu2delta0 met15delta0 ura3delta0*	ATCC
Myo1-GFP	*MATa leu2delta0 met15delta0 ura3delta0 MYO1-(GFP-His3MX6)*	ThermoFisher
Myo1-TAP (orf)-HA	*MATa leu2delta0 met15delta0 ura3delta0 MYO1*::*(CBP-*	This study
	*TEV -ZZ-His3MX6)* pBG1805-(orf)-His_6_-HA-3C-ZZp	

### Bait construction

**Figure 1:**
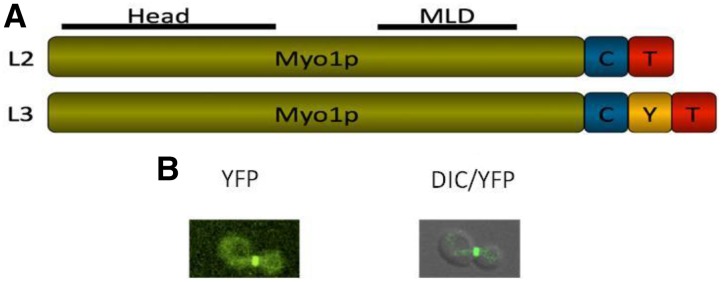
(A) Bar diagram representing the expressed Myo1p bait with iMYTH tags integrated by homologous recombination at the C-terminus. The iMYTH tag consists of the C-terminal half of ubiquitin (Cub; labeled as C), and a transcription factor (labeled as T) comprised of the *Escherichia coli* LexA DNA-binding protein, and the herpes simplex virus VP16 transcriptional activation domain (L2 tag). The L3 variant of the tag contains an YFP molecule (labeled as Y) between C and T. The head domain and the MLD are indicated by lines. (B) *In vivo* localization of the Myo1 bait protein. YFP localization of Myo1p bait protein containing the L3 variant of the tag at the bud-neck in L40 cells. This confirms expression of Myo1p without disruption of function due to mutations and ensures inframe insertion of the CYT (L3) tag.

**Figure 2:**
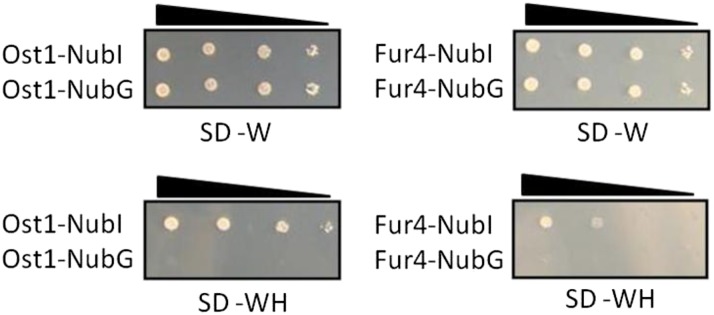
A self-activation test for Myo1p bait construct. The Myo1p bait is intact and does not self-activate. Interactions and growth on selective medium composed of synthetic dropout medium containing 2% dextrose, and lacking both tryptophan and histidine (SD–WH) (bottom two panels), is observed only using the positive control NubI-fusions to the Ost1 and Fur4 proteins. Negative control preys, Ost1 and Fur4 proteins fused to NubG did not show interaction and growth on selective SD–WH medium. All transformed cells grew on SD–W media (synthetic dropout medium containing 2% dextrose and lacking tryptophan) (top panels), which selects only for the presence of control plasmids (and not interaction). Each row represents an individual colony of the Myo1p L40 L2 strain transformed with the indicated control prey plasmid. Colonies were resuspended in 0.9% NaCl, serially diluted (left to right = 10^0^, 10^−1^, 10^−2^, and 10^−3^), and spotted onto selective medium.

To construct the bait strain for iMYTH experiments, we followed the protocol described by Snider *et al.* 2010. Briefly, to generate Myo1 L40 L2 and Myo1 L40 L3-containing strains (Figure 1A), the Myo1-MYTH 5′ (CGAAAAATATTGATAGTAACAATGCACAGAGTAAAATTTTCAGTATGTCGGGGGGATCCCTCC) and Myo1-MYTH 3′ (GGTGAAAGAGTTCATGCCACTTAGTATATAACGCTCGTGTCGTCACTATAGGGAGACCGGCAG) primers were used to PCR-amplify a Cub-LexA-VP16 KanMX, or Cub-YFP-LexA-VP16 KanMX, cassette from L2 or L3 plasmids, respectively (Snider *et al.* 2010). The L40 yeast reporter strain was transformed with the cassette, and transformants were selected with YPD + 200 μg/mL G418. For bait validation, fluorescence microscopy (Figure 1B) and NubG/I self-activation tests (Figure 2) were performed.

### Library transformation, bait-dependency test, and interactome generation

Myo1 L40 was transformed with NubG-X genomic and cDNA libraries provided by the Stagljar laboratory. Positive clones were selected using synthetic dropout medium without tryptophan (SD–W). The plasmids recovered from the transformed yeast cells were partially sequenced (∼300 bp) to identify the encoded gene, and their interaction with the bait protein was reconfirmed using a bait dependency test under selection using synthetic dropout medium without tryptophan and histidine (SD–WH) (Snider *et al.* 2010). The exact size of the prey cDNAs was not determined. The A0287 strain used as a negative control in the bait dependency test expresses a Matα signal sequence and the transmembrane domain of human T-cell surface glycoprotein CD4 fused to the C-terminal ubiquitin domain plus transcription factors LexA and VP16. The positive interactors were classified according to their Gene Ontology (GO; http://geneontology.org/) functions and previously reported physical and genetic interactions. The Biological General Repository for Interaction Datasets (BioGRID; http://thebiogrid.org/), a curated database of yeast protein–protein and genetic interactions, was used to identify previously reported interactions. Cytoscape (http://www.cytoscape.org/) was used to generate two-dimensional interaction maps for visualization of these interactions.

### In vitro validation of the Myo1p iMYTH interactors by coimmunoprecipitation

To capture the Myo1p-interacting proteins for *in vitro* validation of the physical interactions identified by iMYTH, and for their subsequent identification by coimmunoprecipitation (coIP), we employed a modified pull-down approach (Babu *et al.* 2009) using a TAP epitope fused to the Myo1p C-terminus (Myo1-TAP). The Myo1-TAP bait strain was transformed with a full-length prey protein in expression vector BG1805, a URA3 multicopy 2 μ plasmid containing a GAL1 promoter (Gelperin *et al.* 2005), using the standard lithium acetate procedure (Gietz and Woods 2006). The Myo1 protein was captured using the calmodulin-binding peptide in the TAP epitope. Briefly, 40 ml of culture was grown for 2 d in YPD at 30° at 200 rpm. Subsequently, the cells were centrifuged at 3000 rpm for 3 min, and washed twice with 20 ml IPLB buffer (20 mM Hepes KOH, pH 7.4, 150 mM KOAc, 2 mM Mg(Ac)_2_, 2 mM CaCl_2_, and 10% glycerol). For plasmid expression, cells were resuspended in YP-GAL, and incubated for 2–4 hr. The yeast cells were lysed with breaking buffer (IPLB buffer plus 1% Triton-X100 and 1× Protease inhibitor cocktail 1 from Calbiochem (EMD, #539131), and disrupted with glass beads by vortexing at maximum speed for 5 min. Cell lysates were cleared by centrifugation at 4000 rpm for 4 min. For protein isolation, 50 μL of calmodulin beads were mixed with 700 μL of the cell lysate in a microcentrifuge tube, and placed in a rotator for 2 hr at 4°. After incubation, the beads were centrifuged at 5000 rpm for 2 min at 4°. The resulting supernatant was removed and the beads were washed six times with 800 μL of cold IPLB. The beads were then resuspended in an equal volume of IPLB and denatured with 2× Laemmli dye at 95°. The denatured proteins were electrophoresed in a 10% SDS-PAGE gel, and western blot analysis was performed using anti-HA probe (Y-11; Santa Cruz Biotechnology). HRP-anti-rabbit antibody and enhanced chemiluminescent signal detection reagents (Pierce) were used to visualize the prey protein bands.

### In vitro validation of the Myo1p iMYTH interactors by affinity-purification-mass spectrometry (AP-MS)

The affinity capture of GFP tagged to the Myo1 C-terminus (Myo1-GFP), and subsequent analysis by MS was also used for *in vitro* validation of iMYTH Myo1p interacting proteins. Briefly, 40 ml of Myo1-GFP and BY4741 cultures were grown overnight in YPD. The cells were harvested at 3000 rpm for 5 min. The pellet was resuspended in 1 ml IPLB + 1× Protease inhibitor cocktail 1. The cells were disrupted via 5 min vortexing with an equal volume of glass beads. The supernatant was added to 50 µL Miltenyi µbeads (Anti-GFP, #130-094-252), and incubated on ice with rotation for 30 min.

Miltenyi magnetic bead columns were washed prior to use with 200 µL IPLB. Next, the lysate was added, and the beads subsequently washed three times with 800 µL Native IP buffer (50 mM Tris, pH 7.5; 150 mM NaCl; 2.5 mM EDTA), and two times with 500 µL of the same buffer. The bound proteins were released with 100 μL of elution buffer (2 M Urea, 50 mM Tris, pH 7.5, 5 mM Chloracetamide). Tryptic digestion for MS was performed with 5 μg/ml Trypsin gold (Promega, #V5280). The peptides were selected and desalted, using ZipTip with 0.2 µL C_18_ resin (Zip tips, EMD Millipore #ZTC18M960). The protein isolations were conducted in triplicate. Each sample was subjected to analysis with an Orbitrap Elite mass spectrometer to generate MS/MS spectra. The results obtained were matched against a yeast protein sequence database using SEQUEST search engine and scored according to the number of unique peptides identified and the STATQUEST algorithm. Matches were considered valid if they contained more than two unique peptide fragments and matches of > 95% probability.

## Results and Discussion

The iMYTH system is a powerful tool for screening protein–protein interactions, both *in vivo* and in the natural cellular environment where these interactions are expected to occur (Paumi *et al.* 2007; Snider *et al.* 2010). The advantage of iMYTH is that it may detect weak protein–protein interactions, and/or temporally regulated interactions, which may not be detected by conventional pull-down methods. The *S. cerevisiae* wild-type myosin type II (Myo1p) localizes to the bud-neck during cell division (Bi *et al.* 1998); the Myo1p fusion protein used as the bait in this study was also shown to localize precisely to this site (Figure 1B). Another advantage of iMYTH is that the bait, in this case the Myo1 fusion protein, is localized in its natural cellular environment instead of an artificial one, such as the nucleus, as in other classical yeast two-hybrid systems (Drees *et al.* 2001). Another valuable feature of the iMYTH system is that the bait protein is expressed by its native promoter so that the protein levels are not artificially elevated. Furthermore, the system is monitored for self-activation of the bait modules through the use of positive and negative control preys (Figure 2), helping reduce the incidence of false positive results.

**Figure 3:**
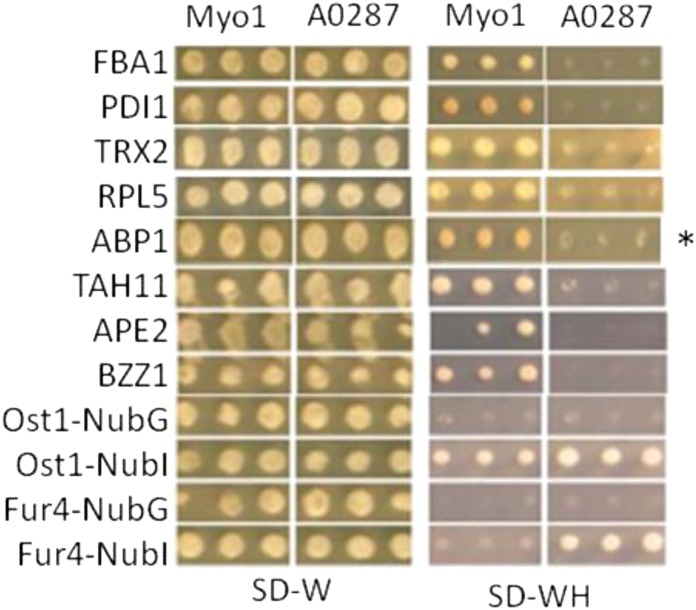
Confirmed iMYTH Myo1p hits. The top eight out of 30 confirmed iMYTH hits are shown. The remaining hits have been included in Figure S1. In the top eight rows, growth of Myo1 L40 strain containing the prey protein was observed on synthetic dropout medium containing 2% dextrose and lacking both tryptophan and histidine (SD–WH), while no growth was observed for corresponding A0287 strain, thereby confirming the specificity of each bait–prey interaction. The asterisk identifies Abp1p, the prey protein confirmed by AP-MS. The remaining 7 iMYTH hits were confirmed by coIP. The bottom four rows show growth in Ost1-NubI and Fur4-NubI positive control preys (described in Figure 2).

In this study, we used a modified iMYTH screen to identify Myo1p-interacting proteins. A total of 30 initial hits were identified (Supplemental Material, Figure S1), all of which passed a secondary bait dependency test. Eight of these hits were confirmed by coIP or AP-MS (Figure 3, Table 2, and Table 3, respectively), and were therefore considered as highly reliable Myo1p-interacting proteins. A null mutation of *ABP1* did not visibly alter the bud neck localization of a Myo1p-YFP fusion protein (data not shown). Haploid null mutations of *FBA1*, *PDI1*, *RPL5*, and *TAH11* are lethal and were not tested. Null strains of *TRX2*, *APE2*, and *BZZ1* were not available for testing.

**Table 2:** Myo1p-interacting proteins validated by coimmunoprecipitation

Gene name	Systematic Name	Description (According to SGD, http://www.yeastgenome.org/)
APE2	YKL157W	Aminopeptidase yscII; may have a role in obtaining leucine from dipeptide substrates; APE2 has a paralog, AAP1, that arose from whole genome duplication
BZZ1	YHR114W	SH3 domain protein implicated in regulating actin polymerization; able to recruit actin polymerization machinery through its SH3 domains; colocalizes with cortical actin patches and Las17p; interacts with type I myosins
FBA1	YKL060C	Fructose 1,6-bisphosphate aldolase; required for glycolysis and gluconeogenesis; catalyzes conversion of fructose 1,6 bisphosphate to glyceraldehyde-3-P and dihydroxyacetone-P; locates to mitochondrial outer surface upon oxidative stress; N-terminally propionylated *in vivo*
PDI1	YCL043C	Protein disulfide isomerase; multifunctional protein of ER lumen, essential for formation of disulfide bonds in secretory and cell-surface proteins, unscrambles non-native disulfide bonds; key regulator of Ero1p; forms complex with Mnl1p, which has exomannosidase activity, processing unfolded protein-bound Man8GlcNAc2 oligosaccharides to Man7GlcNAc2, promoting degradation in unfolded protein response; PDI1 has a paralog, EUG1, that arose from whole genome duplication
RPL5	YPL131W	Ribosomal 60S subunit protein L5; nascent Rpl5p is bound by specific chaperone Syo1p during translation; homologous to mammalian ribosomal protein L5 and bacterial L18; binds 5S rRNA and is required for 60S subunit assembly
TAH11	YJR046W	DNA replication licensing factor; required for prereplication complex assembly
TRX2	YGR209C	Cytoplasmic thioredoxin isoenzyme; part of thioredoxin system that protects cells against oxidative and reductive stress; forms LMA1 complex with Pbi2p; acts as a cofactor for Tsa1p; required for ER-Golgi transport and vacuole inheritance; with Trx1p, facilitates mitochondrial import of small Tims (Tim9p, Tim10p, and Tim13p) by maintaining them in reduced form; abundance increases under DNA replication stress; TRX2 has a paralog, TRX1, that arose from whole genome duplication

SGD, *Saccharomyces* genome database; ER endoplasmic reticulum.

**Table 3:** Myo1p-interacting proteins validated by affinity purification-mass spectrometry

Gene name	Systematic name	Negative control		MYO1 Replicate 1		MYO1 Replicate 2		MYO1 Replicate 3	
		Total Peptides	Probability (%)	Total Peptides	Probability (%)	Total Peptides	Probability (%)	Total Peptides	Probability (%)
ABP1	YCR088W	2	98.97	3	99.58	5	99.58	4	99.58

Exclusive unique peptide count for new Myo1p interactors identified by iMYTH and validated by affinity purification-mass spectrometry.

**Figure 4:**
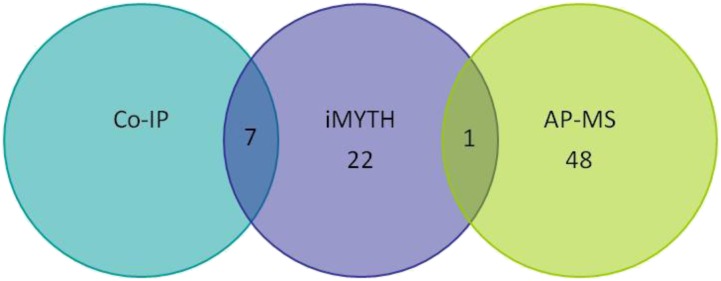
Venn diagram showing the 30 novel Myo1p-interacting partners identified by iMYTH, AP-MS, and coIP. These 30 Myo1p-interacting proteins (purple) have not been reported previously among the 103 Myo1p interactors listed in the SGD. Seven of the 30 novel iMYTH-positive interactors were validated by coIP (teal); one of the 30 was validated by AP-MS experiments (green). None of these Myo1p-interacting proteins were validated by all three methods. Therefore, a total of eight were classified as confirmed positive Myo1p interactors.

The high number of validated hits identified (26.7%) demonstrated the value of iMYTH as a robust high-throughput screening tool for the discovery of novel target proteins with important regulatory functions. None of the 30 Myo1p iMYTH interactors identified in this study (Figure S1) were previously reported in the *Saccharomyces* Genome Database (SGD; http://www.yeastgenome.org/) (see summary in Figure 4). Furthermore, we did not identify other expected interactors in this screen that were previously reported in the SGD, such as actin (*ACT1*), or the myosin light chains (*MLC1* and *MLC2*) (Drees *et al.* 2001; Gavin *et al.* 2002; Krogan *et al.* 2006). This may have been due to the physical constraints of the iMYTH construct, where *ACT1* interacts with the actin-binding domain at the N-terminal region of Myo1p, while *MLC1* and *MLC2* bind to the neck region proximal to the ATPase domain of Myo1p. Nonetheless, when the *ACT1* gene was cloned in the prey plasmid BG1805, and coexpressed with the Myo1p bait, it was confirmed to interact with Myo1p in coIP assays (Figure S2A). Therefore, the absence of clones encoding Act1p, and possibly other known Myo1p-interacting proteins, in our iMYTH screen may have been caused by a low representation of these clones in the prey library. Cloning and expression of the individual genes in a prey vector is therefore the recommended approach to validate these and other reported Myo1p-interacting proteins. On the other hand, other known interactors of Myo1p that were missed, such as Mlc1p, Mlc2p, Bni5p, and Kar2p, as well as Act1p, were reconfirmed by our AP-MS experiments (Table 4). Therefore, the iMYTH method was capable of identifying different types of protein–protein interactions that are not stabilized under the conditions employed in traditional capture methods such as AP-MS. In this regard, the coIP assay strategy used here, coupled with detection by Western blot, proved to be a more effective method for confirmation of iMYTH hits.

**Table 4:** Exclusive unique peptide count for Myo1p physical interactors previously reported in the SGD and confirmed by affinity purification-mass spectrometry in this study

Gene name	Systematic name	Negative control		MYO1 Replicate 1		MYO1 Replicate 2		MYO1 Replicate 3	
		Total Peptides	Probability (%)	Total Peptides	Probability (%)	Total Peptides	Probability (%)	Total Peptides	Probability (%)
MLC1	YGL106W	0	0	15	99.58	6	99.58	4	86.03
MLC2	YPR188C	0	0	33	99.58	41	99.58	32	99.58
BNI5	YNL166C	1	96.08	2	99.58	3	99.58	5	99.58
KAR2	YJL034W	2	96.57	2	96.12	5	99.58	3	98.17
ACT1	YFL039C	5	99.58	34	99.58	17	99.58	14	99.58

**Figure 5:**
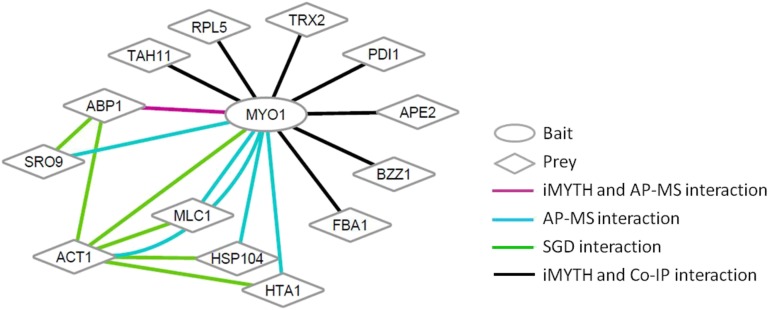
Proposed network of Myo1p-interacting proteins identified by iMYTH, coIP, and AP-MS. A Myo1-TAP construct was used as the bait protein to confirm seven novel Myo1p-interacting proteins by subsequent coIP assays. Abp1p, also a novel interactor, was confirmed by AP-MS as listed in Table 2 and Table 3, respectively. Previously reported physical interactors of Myo1p: Mlc1p, Mlc2p, Bni5p, Kar2p, and Act1p were confirmed by AP-MS (see Table 4).

AP-MS analysis of Myo1p-GFP pull downs—a method capable of pulling down large protein complexes attached to Myo1p irrespective of their site of interaction within the protein—generated 49 Myo1p physical interactors with >2 unique (present at least in one of the three replicate purifications), statistically significant peptides (>90%) (Table S1). Bni5, a septin protein required for cytokinesis in yeast (Lee *et al.* 2002) that was previously reported to interact with Myo1p (Schneider *et al.* 2013), is among the five proteins reconfirmed by our study (Table 4). To summarize our findings, a proposed interaction network incorporating the eight novel Myo1p-interacting proteins confirmed in this study is presented in Figure 5.

Abp1p is an actin-binding protein of the cortical actin cytoskeleton that is important for activation of the Arp2/3 complex that plays a key role in actin cytoskeleton organization and inhibits barbed-end actin filament elongation (Michelot *et al.* 2013). Given its known function in organization of the actin cortical cytoskeleton, we propose that Abp1p could be associated with the process of disassembly of the actin ring as it contracts during cytokinesis. The confirmation of Bzz1p as a Myo1p-interacting protein by both iMYTH and coIP places another protein implicated in regulating actin polymerization (Soulard *et al.* 2002), together with Myo1p potentially at the bud neck. These results support the possibility that Abp1p and Bzz1p with Bni5p (as reported previously by Schneider *et al.* 2013) may function together with Myo1p as part of a complex to regulate actin filament dynamics at the cytokinetic ring. Going forward, it will be valuable to map the sites of iMYTH interaction between Myo1p and the recovered protein set, and to investigate the functional consequences of direct and indirect associations revealed by this study.

## Supplementary Material

Supplemental Material
