# Supplementary material for: Novel Interactome of Saccharomyces cerevisiae Myosin Type II Identified by a Modified Integrated Membrane Yeast Two-Hybrid (iMYTH) Screen
Source: G3 (Bethesda). 2016 Feb 25;6(5):1469–74. doi: 10.1534/g3.115.026609 (PMC4856097; doi:10.1534/g3.115.026609)
Supplement: Supplemental Material [file supp_6_5_1469__index.html]

Novel Interactome of Saccharomyces cerevisiae Myosin Type II Identified by a Modified Integrated Membrane Yeast Two-Hybrid (iMYTH) Screen — Novel Interactome of Saccharomyces cerevisiae Myosin Type II Identified by a Modified Integrated Membrane Yeast Two-Hybrid (iMYTH) Screen — Supplemental Material 

# Novel Interactome of *Saccharomyces cerevisiae* Myosin Type II Identified by a Modified Integrated Membrane Yeast Two-Hybrid (iMYTH) Screen

## Supplemental Material for Santiago *et al.*, 2016

**Files in this Data Supplement:**

- Figure S1 - Myo1p hits identified in iMYTH experiments. (.pdf, 277 KB)
- Figure S2 - Co-immunoprecipitation validation of iMYTH Myo1 interactions. (.pdf, 352 KB)
- Table S1 - Exclusive, unique peptide count for all Myo1p-interacting proteins identified by affinity purification-mass spectrometry. (.pdf, 392 KB)
