# Supplementary material for: Novel Interactome of Saccharomyces cerevisiae Myosin Type II Identified by a Modified Integrated Membrane Yeast Two-Hybrid (iMYTH) Screen
Source: G3 (Bethesda). 2016 Feb 25;6(5):1469–74. doi: 10.1534/g3.115.026609 (PMC4856097; doi:10.1534/g3.115.026609)
Supplement: Supplemental Material [file supp_g3.115.026609_FigureS1.pdf]

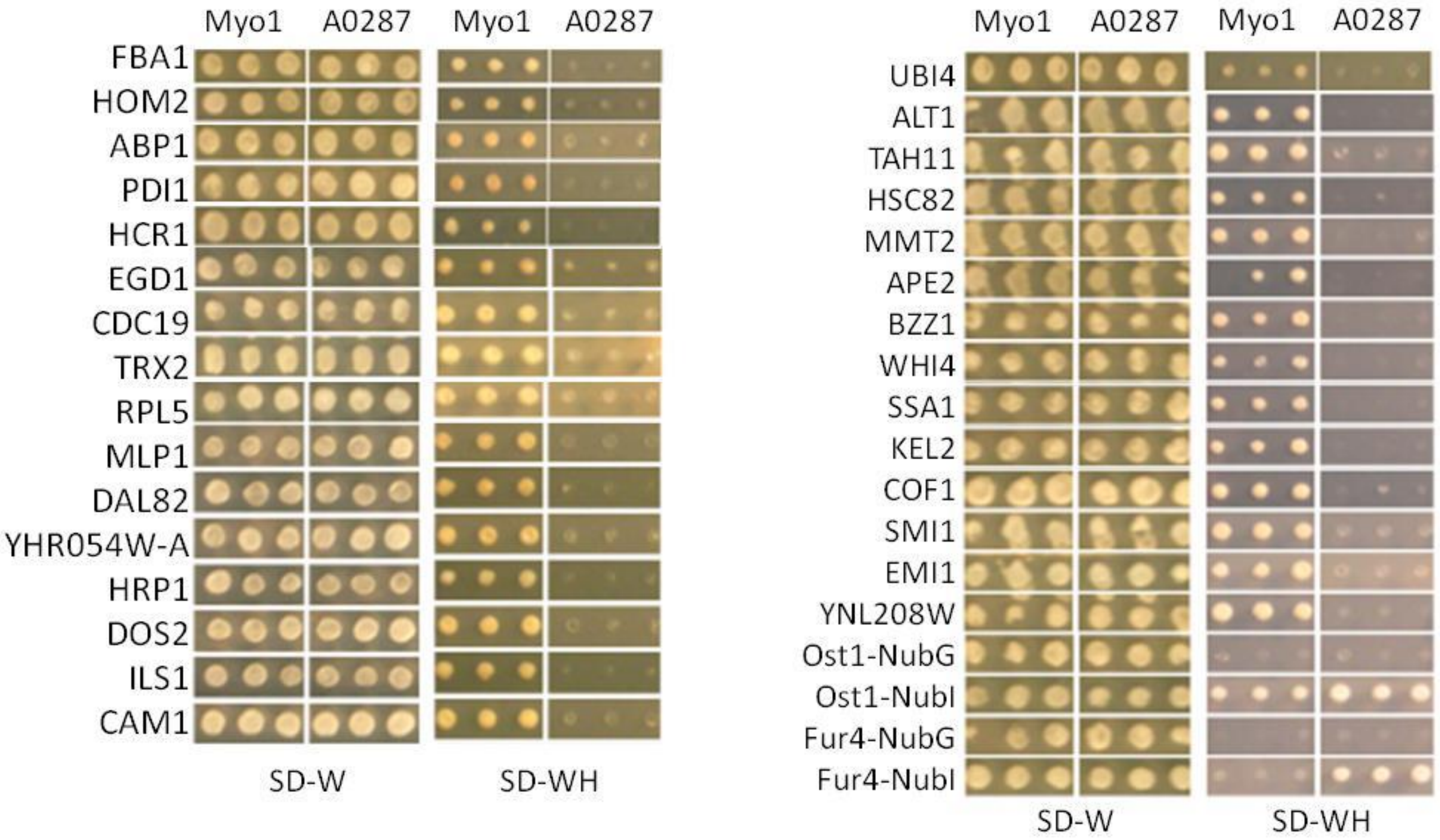

Figure S1. Myo1p hits identified in iMYTH experiments. Plasmids were extracted from positive iMYTH clones, transformed into the Myo1 L40 (Myo1) or Artificial bait strain (A0287), and cultured under selective conditions (SD-W plasmid selection medium and SD-WH interaction selection medium) Ost1-NubG and Fur4-NubG were used as negative prey plasmids. Ost1-NubI and Fur4-NubI were used as positive prey plasmids.
