# Supplementary material for: Novel Interactome of Saccharomyces cerevisiae Myosin Type II Identified by a Modified Integrated Membrane Yeast Two-Hybrid (iMYTH) Screen
Source: G3 (Bethesda). 2016 Feb 25;6(5):1469–74. doi: 10.1534/g3.115.026609 (PMC4856097; doi:10.1534/g3.115.026609)
Supplement: Supplemental Material [file supp_g3.115.026609_FigureS2.pdf]

A

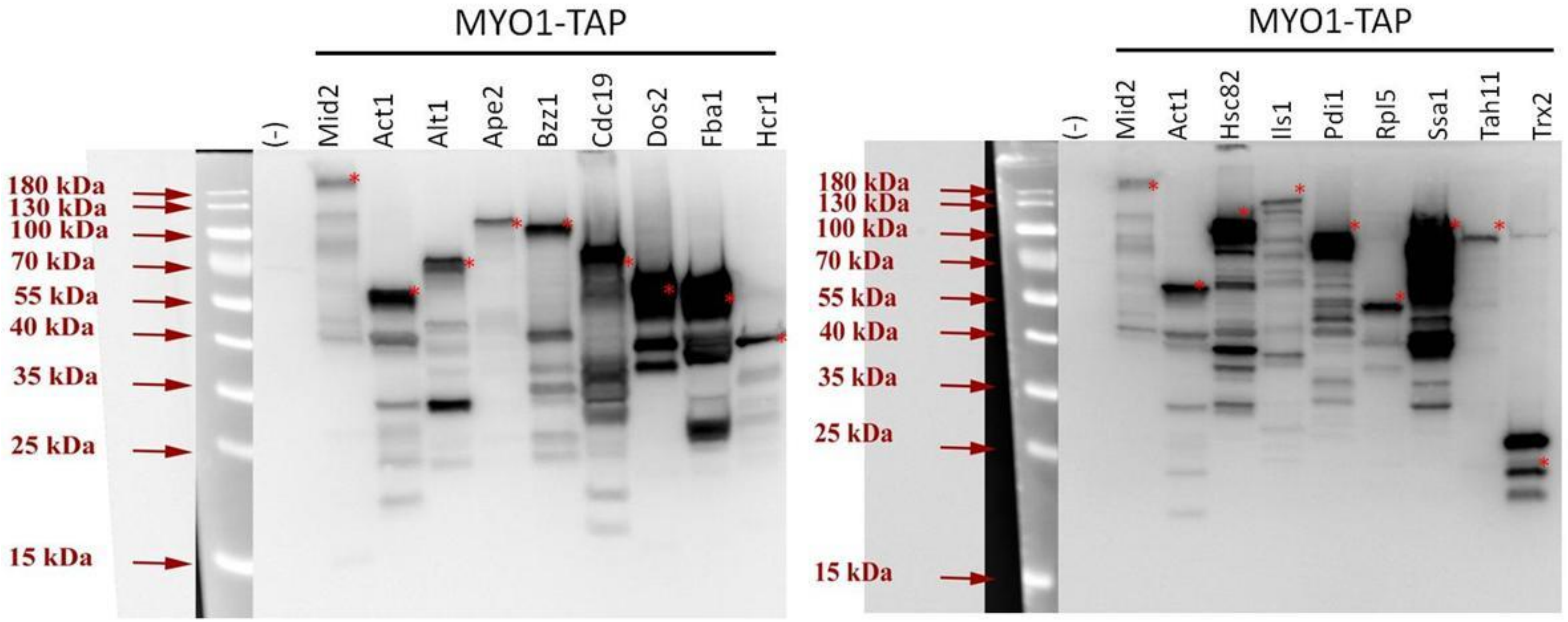
MYO1-TAP
(-)
Mid2
Act1
Alt1
Ape2
Bzz1
Cdc19
Dos2
Fba1
Hcr1
180 kDa
130 kDa
100 kDa
70 kDa
55 kDa
40 kDa
35 kDa
25 kDa
15 kDa
MYO1-TAP
(-)
Mid2
Act1
Hsc82
Ils1
Pdi1
Rpl5
Ssa1
Tah11
Trx2
180 kDa
130 kDa
100 kDa
70 kDa
55 kDa
40 kDa
35 kDa
25 kDa
15 kDa

B

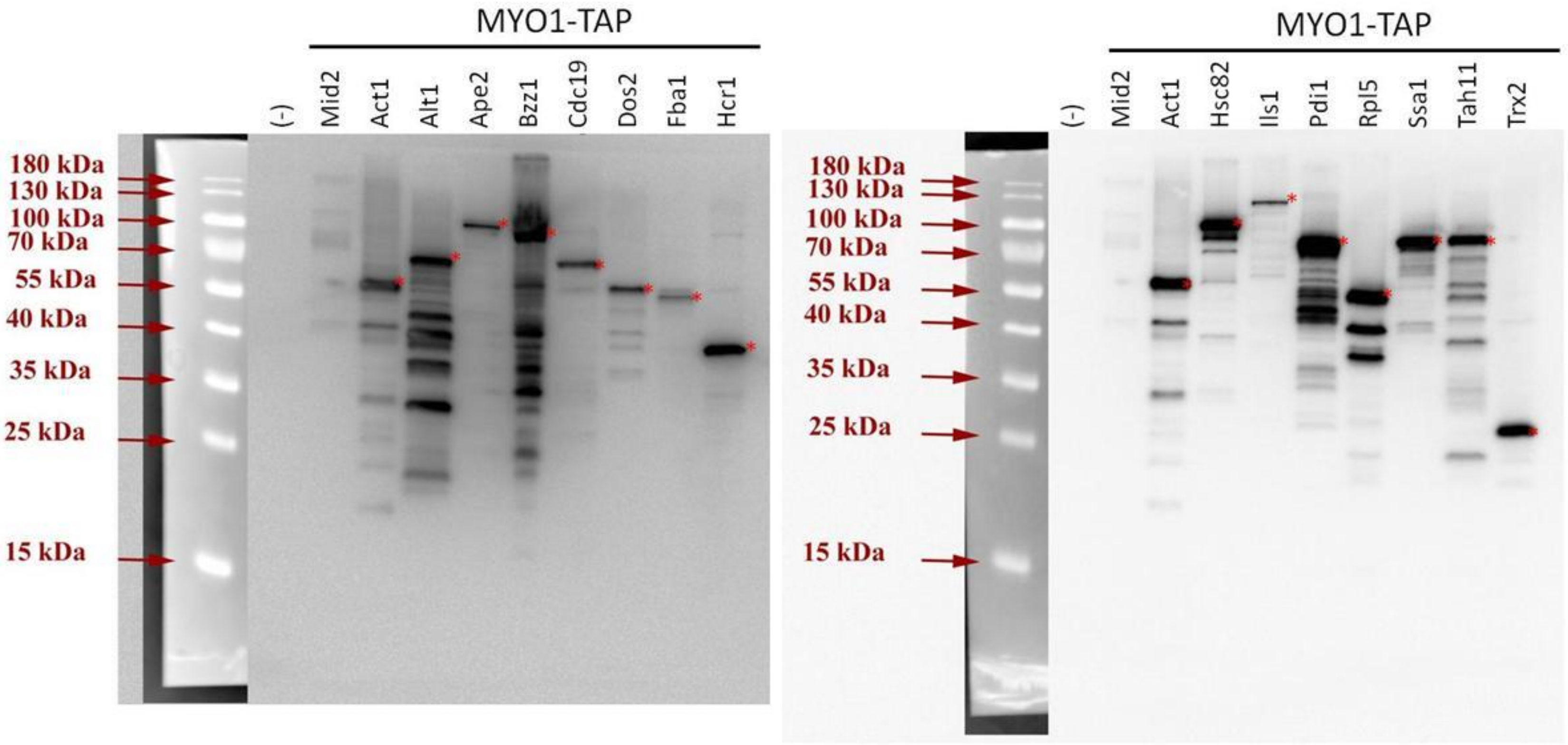

MYO1-TAP
(-)
Mid2
Act1
Alt1
Ape2
Bzz1
Cdc19
Dos2
Fba1
Hcr1
180 kDa
130 kDa
100 kDa
70 kDa
55 kDa
40 kDa
35 kDa
25 kDa
15 kDa
MYO1-TAP
(-)
Mid2
Act1
Hsc82
Ils1
Pdi1
Rpl5
Ssa1
Tah11
Trx2
180 kDa
130 kDa
100 kDa
70 kDa
55 kDa
40 kDa
35 kDa
25 kDa
15 kDa

C

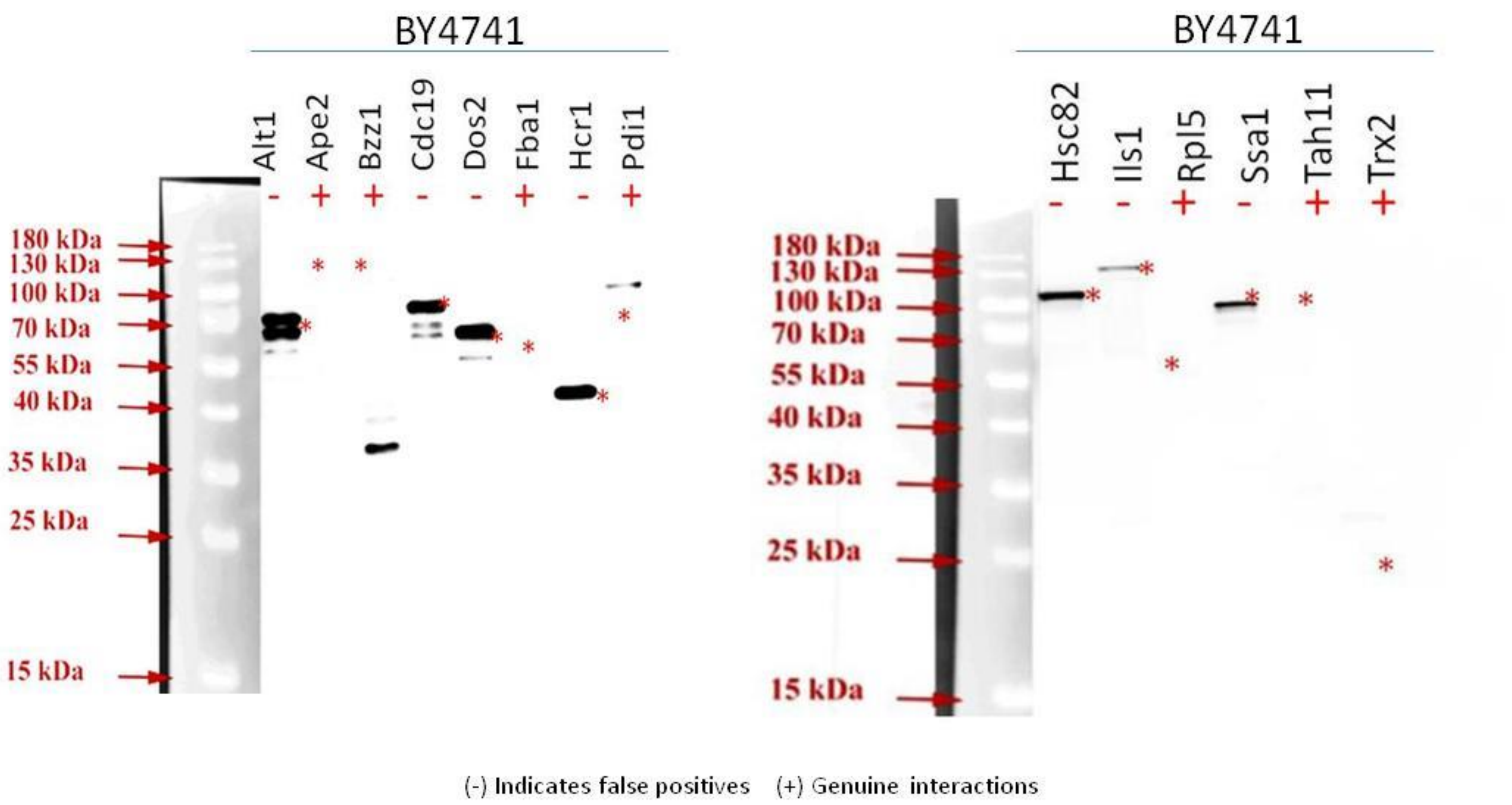

Figure S2. Co-immunoprecipitation validation of iMYTH Myo1 interactions. Validation of Myo1-iMYTH interactions by co-immunoprecipitation (co-IP) is shown. (A) Whole cell lysates without co-IP. (B) co-IP experiments. (C) HA-tagged constructs in the untagged wild-type strain (BY4741). The expected size of prey protein plus ~ 19kDa of the tandem fusion tag is indicated by an asterisk. The untagged strain was used as a negative control (-) in A and B. Mid2 and Act1 prey proteins were used as negative and positive controls, respectively.
