# Supplementary material for: Novel Interactome of Saccharomyces cerevisiae Myosin Type II Identified by a Modified Integrated Membrane Yeast Two-Hybrid (iMYTH) Screen
Source: G3 (Bethesda). 2016 Feb 25;6(5):1469–74. doi: 10.1534/g3.115.026609 (PMC4856097; doi:10.1534/g3.115.026609)
Supplement: Supplemental Material [file supp_g3.115.026609_TableS1.pdf]

Table S1. Exclusive, unique peptide count for all Myo1p-interacting proteins identified by affinity purification-mass spectrometry.

Gene name	Systematic name	Negative control		MYO1 Replicate 1		MYO1 Replicate 2		MYO1 Replicate 3	
		Total Peptides	Probability (%)	Total Peptides	Probability (%)	Total Peptides	Probability (%)	Total Peptides	Probability (%)
MYO1	YHR023W	0	0	433	99.58	387	99.58	292	99.58
MLC2	YPR188C	0	0	33	99.58	41	99.58	32	99.58
MLC1	YGL106W	0	0	15	99.58	6	99.58	4	86.03
RPS12	YOR369C	0	0	12	99.58	4	99.47	11	99.58
ATP1	YBL099W	0	0	10	99.58	5	99.58	7	99.58
ADH1	YOL086C	0	0	7	99.58	15	99.58	5	99.58
MAK21	YDR060W	0	0	6	99.58	5	99.58	3	99.58
ATP2	YJR121W	0	0	6	99.58	3	99.58	3	99.58
YNL050C	YNL050C	0	0	5	99.58	1	99.58	4	99.58
RTN1	YDR233C	0	0	4	99.58	5	99.58	9	99.58
ALD4	YOR374W	0	0	4	99.58	5	99.58	1	99.58
SAS10	YDL153C	0	0	3	99.58	1	99.58	1	98.41
MSS116	YDR194C	0	0	3	99.58	1	99.58	8	99.58
NCE102	YPR149W	0	0	3	97.95	3	97.3	4	99.44
HTA1	YDR225W	0	0	2	95.8	2	99.58	3	99.24

RGI2	YIL057C	0	0	2	99.58	2	99.58	2	99.58
DRS1	YLL008W	0	0	2	96.12	2	99.44	2	99.58
RGC1	YPR115W	0	0	2	99.58	2	99.58	3	99.58
OLA1	YBR025C	0	0	1	91.04	2	98.06	2	96.26
HSP104	YLL026W	0	0	1	98.78	1	92.59	3	99.58
PUB1	YNL016W	0	0	1	97.94	2	92.33	1	96.16
RPA190	YOR341W	0	0	1	99.58	3	99.58	3	99.58
RPL6A	YML073C	1	62.22	7	93.77	6	99.44	3	99.44
NOP13	YNL175C	1	79.25	6	99.58	6	96.49	4	95.8
CIC1	YHR052W	1	99.58	5	99.58	2	99.58	5	99.58
TMA23	YMR269W	1	70.96	5	99.58	4	99.58	5	99.58
KRI1	YNL308C	1	96.1	4	99.58	5	99.58	1	99.58
ENP1	YBR247C	1	51.31	3	99.58	3	99.58	4	99.58
STI1	YOR027W	1	85.68	3	99.58	2	98.11	3	99.58
RPL31A	YDL075W	1	74.53	2	99.58	2	96.6	5	99.58
NUG1	YER006W	1	99.52	2	99.58	2	99.58	5	99.58
RPS30A	YLR287C-A	1	86.26	2	99.43	2	99.47	1	97.94
BNI5	YNL166C	1	96.08	2	99.58	3	99.58	5	99.58
RRP5	YMR229C	2	99.58	15	99.58	14	99.58	14	99.58

YGR283C	YGR283C	2	99.58	11	99.58	4	99.58	10	99.58
RPL30	YGL030W	2	99.58	8	99.58	4	99.58	2	99.58
GPM1	YKL152C	2	99.44	6	99.21	12	99.58	3	97.63
RPL22A	YLR061W	2	99.58	6	99.58	4	99.58	5	99.58
BMS1	YPL217C	2	99.58	5	99.58	1	99.58	5	99.58
PCK1	YKR097W	2	99.24	4	99.58	17	99.58	6	99.58
SAM1	YLR180W	2	99.58	4	99.58	3	99.58	5	99.58
RPS1B	YML063W	2	92.01	4	99.56	4	99.58	2	91.49
RPL36A	YMR194W	2	98.17	4	98.47	4	99.58	2	97.63
PFK2	YMR205C	2	99.58	4	99.58	3	99.58	3	99.58
SRO9	YCL037C	2	99.58	3	99.58	2	99.58	7	99.58
KRR1	YCL059C	2	99.58	3	99.58	3	99.58	3	99.58
ABP1	YCR088W	2	98.97	3	99.58	5	99.58	4	99.58
PMR1	YGL167C	2	99.58	3	99.58	7	99.58	5	99.58
KAR2	YJL034W	2	96.57	2	96.12	5	99.58	3	98.17
SSE1	YPL106C	2	91.5	1	96.39	1	96.49	2	99.58
